# Supplementary material for: Neural network-based method to stratify people at risk for developing diabetic foot: A support system for health professionals
Source: PLoS One. 2023 Jul 13;18(7):e0288466. doi: 10.1371/journal.pone.0288466 (PMC10343027; doi:10.1371/journal.pone.0288466)
Supplement: Attachment — Submitted filename: Response to Reviewers.docx [file pone.0288466.s001.docx]

**PONE-D-22-26803**

Imran Ashraf, Ph.D.

Academic Editor

PLOS ONE

Manuscript: **PONE-D-22-26803**

Title: **Neural network-based method to stratify people at risk for developing diabetic foot: a support system for health professionals**

Dear Editors,

The authors appreciate the valuable comments. We have submitted the revised version of the paper, following the reviewers’ suggestions. A point-to-point list of the amendments is appended below. In order to make it easier to follow our responses, we have repeated each of the reviewers’ comments before providing the changes we have made in the manuscript in blue. In the revised manuscript, we have highlighted all changes and improvements in red color.

Best regards,

Danton Diego Ferreira

**Answers to the Reviewers**

**Reviewer #1:** The review of the paper entitled “Neural network-based method to stratify people at risk for developing diabetic foot: a support system for health professionals” Background and Objective: Diabetes Mellitus (DM) is a chronic disease with a high prevalence worldwide. Diabetic foot is one of the DM complications and compromises health and quality of life, due to the risk of lower limb amputation. This work is aimed to build a risk classification system for the development of the diabetic foot, using Artificial Neural Networks (ANN). Methods: It is a methodological study in which two databases were used, one for system design (training and validation) containing 250 people with DM and another for testing, containing 141 people. Each person answered a questionnaire with 54 questions about foot care and sociodemographic information. People from both databases were classified by specialists as high or low risk for diabetic foot. Supervised ANN (multi-layer Perceptron - MLP) models were exploited and a smartphone app was built. The app returns a personalized report indicating self-care for each user. System Usability Scale (SUS) was used for the usability evaluation.

I have found the following concerns which need to correct…

1. The introduction part is in multiple paragraphs, please rearrange the article text. Arrange multiple paragraphs in one if applicable.

Thank you for this suggestion. We have rearranged the introduction section in which some paragraphs were arranged in one.

2. I never found any novel work that is highlighted.

We have rewritten this sentence in the new manuscript version.

3. Its confusion for readers that what is the data in your article? Still authors talking about the patients, please make sure that which kind of data are you using?

Our database consisted of 54 pieces of information from 250 people with diabetes. These data were collected via questionnaire in a health institution in a city in the state of Minas Gerais, Brazil. The instrument (questionnaire) used for data collection covered the risk factors for the development of diabetic foot proposed by the International Consensus on Diabetic Foot, and by the Primary Care Booklet to Assist People with DM from the Ministry of Health of Brazil, which resulted in a total of 54 variables. Variables related to people's self-care habits concerning their health, foot care, perceived changes in the feet, as well as sociodemographic and socioeconomic variables were included.

This information is in the first two paragraphs of Section 2. To make this part clearer, we rewrote the first paragraph.

4. ANN is a very much older method, if authors try to apply any deep learning method, it could be helpful.

In this paper we used a multi-layer perceptron (MLP), which is the most popular type of artificial neural network (ANN). An MLP is an ANN capable of handling both linearly separable and non-linearly separable data. It belongs to a class of neural networks known as feed-forward neural networks, which connect the neurons in one layer to the next layer in a forward manner. It consists of interconnected neurons which process data through three or more layers. The basic structure of an MLP consists of an input layer, one or more hidden layers and an output layer. There is no restriction on the number of hidden layers, however, an MLP usually has a small number of hidden layers. In our work we used only one hidden layer. Due to their simplicity, MLPs usually require short training times to learn the representations in data and produce an output.

A Deep Neural Network (DNN) is simply an artificial neural network with deep layers. Deep layers in this context mean that the network has several layers stacked together for processing and learning from data. It is important to note that an MLP is considered an example of DNNs. Due to their complex nature, DNNs usually require long periods of time to train the network on the input data. Additionally, they require powerful computers with specialized processing units such as Tensor Processing Units (TPU) and Neural Processing Units (NPU).

It is possible that the use of a DNN instead of the simple MLP employed may lead to a model with slightly higher accuracy. On the other hand, it will require more data to be trained, a specialized processing unit, and probably would result in a more complex model. As we discussed in Section 5.1, we opted by more parsimonious models, and, in this case, the MLP is more appropriated than a DNN.

5. Authors only talking about the ANN is different forms, and try to change the dataset but not the original method. But still readers are unaware of data, what is the input to the system?

To make the data description better, we have rewritten section 2 so that more details about the variables and their codification was added in the new manuscript version.

In addition, we included paragraph 4 in Section 4 of the new manuscript version, where we described the MLP inputs for each approach developed.

6. Figure one and other figures are not according to the standard. It should be 300dpi.

The figures were edited accordingly.

7. Please add more recent papers in the references.

He revised the paper and included seven new references of the last two years.

8. Try to compare your work with others in the same domain.

In Section 5.2, Table 3, of the submitted manuscript version, we have already presented a comparison between our best model and a Competitive Neural Layer model proposed in reference [11] for the same purpose. In this case, the model proposed in [11] was implemented and applied to the same testing data set. In addition, in the Discussion subsection we have related our achievements with other works in the same domain.

**Reviewer #2:** The manuscript focuses on detecting Diabetes Mellitus (DM) by using AI technique. Reducing the contributory reasons of DM from 54 to 10, appears to be a contribution of this paper. Although, with the available compute power today, processing 54 attributes for class identification is not a challenge, however, from an end-user perspective, data collection against 10 questions would be more efficient. As a suggestive added check, identification of most important parameters in low risk categorise may be re-validated. The quality of images used in the paper also needs improvement for enhanced visibility.

Thank you for your positive comments and suggestions. We have included a sentence in paragraph five of the new manuscript version to highlight the contribution of the paper in making the data collection easier for the end-user by reducing the contributory reasons of the diabetic foot risk.

Regarding the identification of most important parameters in low risk group, we have rewritten the last paragraph of Section 5.2 to make this part clearer.

The manuscript is written nicely and presents an important issue related to healthcare. The paper is recommended for approval.

Thank you for the positive comments.
