# Supplementary material for: Neural network-based method to stratify people at risk for developing diabetic foot: A support system for health professionals
Source: PLoS One. 2023 Jul 13;18(7):e0288466. doi: 10.1371/journal.pone.0288466 (PMC10343027; doi:10.1371/journal.pone.0288466)
Supplement: Attachment — Submitted filename: Response to Reviewers.docx [file pone.0288466.s002.docx]

**PONE-D-22-26803R1**

Imran Ashraf, Ph.D.

Academic Editor

PLOS ONE

Manuscript: **PONE-D-22-26803R1**

Title: **Neural network-based method to stratify people at risk for developing diabetic foot: a support system for health professionals**

Dear Editors,

The authors appreciate the valuable comments. We have submitted the revised version of the paper, following the reviewers’ suggestions. A point-to-point list of the amendments is appended below. In order to make it easier to follow our responses, we have repeated each of the reviewers’ comments before providing the changes we have made in the manuscript in blue. In the revised manuscript, we have highlighted all changes and improvements in red color.

Best regards,

Danton Diego Ferreira

**Answer to Journal Requirements:**

Please review your reference list to ensure that it is complete and correct. If you have cited papers that have been retracted, please include the rationale for doing so in the manuscript text, or remove these references and replace them with relevant current references. Any changes to the reference list should be mentioned in the rebuttal letter that accompanies your revised manuscript. If you need to cite a retracted article, indicate the article’s retracted status in the References list and also include a citation and full reference for the retraction notice.

We have reviewed the reference list and completed all missing information.

**Answers to the Reviewers**

**Reviewer #1:** Review of the article entitled “Neural network-based method to stratify people at risk for developing diabetic foot: a support system for health professionals”. Diabetes Mellitus (DM) is a chronic disease with a high prevalence worldwide. Diabetic foot is one of the DM complications and compromises health and quality of life, due to the risk of lower limb amputation. This work is aimed to build a risk classification system for the development of the diabetic foot, using Artificial Neural Networks (ANN).

The article has been prepared according to my suggestion. Now have the following minor concerns.

1. Thoroughly check the grammatical errors.

We carefully reviewed the manuscript.

2. Add some references in the background study which improve your paper’s literature review, for example…

10.1109/ACCESS.2019.2896961

10.1109/ACCESS.2021.3056516

https://doi.org/10.3390/app9010069

https://doi.org/10.1155/2022/7954333

https://doi.org/10.3390/math10050796

https://doi.org/10.1155/2022/4942637

The suggested references are related to image processing and segmentation methods based on Deep Learning approaches. Thus, we decided to reference these on the conclusion section where we described our intention for future works.

**Reviewer #3:** In this paper, the authors are proposed “Neural network-based method to stratify people at risk for developing diabetic foot: a support system for health professionals”.

The strengths of the paper are that it is well structured, the description of the related work is well done and that results are extensively compared to results of the similar research.

The authors addressed all the comments. So, i recommend to accept this manuscript..

Thank you for the positive comments.
