# Supplementary material for: Neural network-based method to stratify people at risk for developing diabetic foot: A support system for health professionals
Source: PLoS One. 2023 Jul 13;18(7):e0288466. doi: 10.1371/journal.pone.0288466 (PMC10343027; doi:10.1371/journal.pone.0288466)
Supplement: Attachment — Submitted filename: Response to Reviewers.docx [file pone.0288466.s003.docx]

**PONE-D-22-26803R2**

Imran Ashraf, Ph.D.

Academic Editor

PLOS ONE

Manuscript: **PONE-D-22-26803R2**

Title: **Neural network-based method to stratify people at risk for developing diabetic foot: a support system for health professionals**

Dear Editors,

The authors appreciate the valuable comments. We have submitted the revised version of the paper, following the reviewers’ suggestions. A point-to-point list of the amendments is appended below. In order to make it easier to follow our responses, we have repeated each of the reviewers’ comments before providing the changes we have made in the manuscript in blue. In the revised manuscript, we have highlighted all changes and improvements in red color.

Best regards,

Danton Diego Ferreira

**Answers to the Reviewers**

**Reviewer #1:** Review of the manuscript entitled “Neural network-based method to stratify people at risk for developing diabetic foot: a support system for health professionals”. Diabetes Mellitus (DM) is a chronic disease with a high prevalence worldwide. Diabetic foot is one of the DM complications and compromises health and quality of life, due to the risk of lower limb amputation. This work is aimed to build a risk classification system for the development of diabetic foot, using Artificial Neural Networks (ANN). Methods: It is a methodological study in which two databases were used, one for system design (training and validation) containing 250 people with DM and another for testing, containing 141 people. Each person answered a questionnaire with 54 questions about foot care and socio-demographic information. People from both databases were classified by specialists as high or low risk for the diabetic foot. Supervised ANN (multi-layer Perceptron - MLP) models were exploited and a smartphone app was built. The app returns a personalized report indicating self-care for each user. The system Usability Scale (SUS) was used for the usability evaluation. Results:

I have read the updated paper and still I found some issues which need to be corrected.

1. Discussion is poor, please add a table in the discussion section that should compare your results with other authors in the same domain.

Thank you for your comment. We have included a further discussion at the end of Section 5.1, in which we considered recent works using machine learning approaches to access diabetic foot development. In addition, we have included Table 4 in the revised manuscript, which presents a comparison of methods in terms of feature selection and classification techniques, sample size, clinical exams, number of available variables, number of selected variables, and performance.

2. Please add more recent medical and deep learning research papers in the background section.

https://doi.org/10.1109/ACCESS.2019.2896961

https://doi.org/10.1109/ACCESS.2021.3056516

https://doi.org/10.3390/app9010069

https://doi.org/10.1155/2022/7954333

https://doi.org/10.3390/math10050796

https://doi.org/10.1155/2022/4942637

Thank you for your suggestion, these are interesting papers. We have included more recent medical and deep learning research papers in the Introduction section.

3. Still have a lot of grammatical errors.

We are very sorry for missing these grammatical errors. We sent the revised manuscript for a professional grammar review and hope that there is no further error in this regard.
